# Media Exposure and Media Credibility Influencing Public Intentions for Influenza Vaccination

**DOI:** 10.3390/vaccines10040526

**Published:** 2022-03-29

**Authors:** Chuanlin Ning, Difan Guo, Jing Wu, Hao Gao

**Affiliations:** 1 School of Media and Communication, Shanghai Jiao Tong University, Shanghai 200240, China; ningchuanlin@sjtu.edu.cn; 2 School of Journalism and Communication, Nanjing Normal University, Nanjing 210097, China; guodifan@163.com; 3 Faculty of Social Sciences, University of Ljubljana, 1000 Ljubljana, Slovenia; sunnyjingya@163.com

**Keywords:** technology acceptance model, influenza vaccination, media exposure, media credibility, behavioral intention

## Abstract

Due to the low rate of influenza vaccination in China, this study explores the factors influencing the Chinese public’s influenza vaccination intentions. Based on the technology acceptance model (TAM), this study builds a theoretical model to examine the factors influencing Chinese public intentions toward influenza vaccination. We define media exposure and media credibility as external variables and the perceived characteristics of influenza vaccines as intermediate variables in the proposed model. A total of 597 valid questionnaires were collected online in this study. Combined with structural equation modeling (SEM), SPSS 22.0 and AMOS 17.0 were used to conduct empirical research, supporting the proposed research hypotheses. The results show that media exposure and media credibility have no direct effects on the audience’s intention to take the influenza vaccine. However, media exposure positively influences media credibility, influencing vaccination intentions through perceived usefulness (PU) and perceived ease of use (PEOU). Furthermore, PU and PEOU significantly positively influence behavioral intentions, and PEOU significantly affects PU. This paper has proven that media with better credibility gained more trust from the audience, indicating a new perspective for the promotion of influenza vaccination. This study suggests releasing influenza-related information via media with great credibility, further improving public acceptance of becoming vaccinated.

## 1. Introduction

Influenza has become a significant public health issue to be addressed globally [1]. The vaccine is generally considered one of the most cost-effective ways to avoid disease [2]. However, the WHO listed the ‘global influenza pandemic’ and ‘vaccine hesitation’ among the top 10 global health threats in 2019 [3]. Global vaccination is still a weak link in the progress toward beating influenza. As for influenza vaccination in China, although the national health authority has continuously issued influenza prevention and treatment guidelines, vaccination coverage is still low, at only 2% to 3% per year [4].

There are many reasons for the low vaccination rate and vaccine hesitancy. Studies have shown that media communications correlate with influenza vaccination to some extent. On the one hand, the number of media reports, the timing of reports, and immunization promotion affect the influenza vaccination rate [5]. Traditional mass media, social media, online media, and other media are considered effective methods for promoting influenza vaccination [6,7]. On the other hand, media communications also negatively influence public awareness of the influenza vaccine. Some studies believed that mass media and social media information had become an important factor influencing ‘influenza vaccine hesitancy’ and ‘influenza vaccine panic’ [8,9]. Misleading media information, such as frequent hand washing and eating citrus fruits share the same prevention effectiveness as vaccination, weakens people’s intention to get vaccinated [10]. Meanwhile, some scholars noted that relying on mass media and social media for receiving information against influenza is limited. Family members, health care professionals, and community organizations are also virtual channels [10,11,12].

In summary, influenza has become a global public health issue. As an effective means of prevention and treatment, it is desired to improve public acceptance of influenza vaccination. Researchers have found a correlation between media communications and influenza vaccination [5,6,7,8,9,10]. They believed that media information impacts public awareness of influenza prevention, further affecting influenza vaccination. However, few studies specifically discuss the influence mechanism of media communication, public awareness of the influenza vaccine, and influenza vaccination. Furthermore, research on media communication and influenza vaccination has rarely focused on China. Thus, this paper targets influenza prevention in China, and it builds a model of media communication influencing influenza vaccination based on the technology acceptance model (TAM). To test the model and to explore the influence of media communication on influenza vaccination, we employ structural equation modeling (SEM), which also supports the proposed hypotheses.

## 2. Model and Hypotheses

### 2.1. Technical Acceptance Model (TAM) and Influenza Vaccination

Davis developed the technical acceptance model (TAM) in 1989 to explain the potential behavioral intentions of users when using innovative technologies [13,14,15]. Derived from the theory of reasoned action (TRA) and the theory of planned behavior (TPB) of psychology [16], this model examines the factors influencing users’ behavioral intentions to use new technologies from their perspective [17], and it is a good model for explaining individuals’ motivation to adopt and to use technology [18]. This model proposed two primary factors, ‘perceived usefulness’ (PU) and ‘perceived ease of use (PEOU), that influence users’ intentions toward using the technology: PU refers to whether individuals believe that a particular system can enhance their performance, which directly affects ‘attitude’ and ‘behavioral intention,’ and PEOU refers to an individual defining how easy it is to learn and to use a system, which affects ‘behavioral intention’ through PU. Venkatesh, Viswanath, and Davis argued that the external variables could affect PU and PEOU [19].

Scholars have applied TAM in empirical research and expanded its application in many fields, such as business [20,21,22], government administration [23,24,25], education [26,27,28], media [29,30,31], and medicine [32,33,34]. King and He conducted a statistical meta-analysis of studies using TAM, and they found that TAM had been applied in diverse fields and it was a valid and a robust model with wider potential utility [35]. Specific to vaccination research, Zhao et al. used TAM to explore the factors influencing parents’ intentions to vaccinate their children with the influenza vaccine in urban China [36]. Lu explored the impact of media exposure on intentions to get the HPV vaccine among college students in Guangzhou, Zhuhai, and Macau [32]. Muqattash et al. used TAM as one of their theoretical frameworks to investigate the COVID-19 vaccination preferences of Arab residents [37].

Essentially, the influenza vaccine is a biotechnological invention that helps to protect people from influenza virus infection [38,39]. In terms of the technical process, the development of high-yield vaccine candidates is a complex process involving collaboration between laboratories involved in developing recombinants and WHO Collaborating Centers (CC). Reverse genetics (patented technology) and the classical reassortment (available since 1971) are used currently [38]. Besides, due to variations in influenza viruses, continuous global surveillance of influenza viruses is required to select and to develop the best candidate vaccine viruses for reformulation and production and to supply optimal influenza vaccines [38]. Also, industrial-scale production and accelerated production licensing of influenza vaccines need established, validated, and robust technology [39]. Thus, the development and the production of influenza vaccines require strong technical support and innovation.

Although influenza vaccination is considered to be the most effective means of preventing influenza and its severe consequences [38], influenza vaccine hesitancy still persists [40]. A lack of knowledge about influenza and the technological innovation of the influenza vaccine is an important reason for vaccine hesitancy [41,42], especially the lack of knowledge about the perceived usefulness and the need for influenza vaccination [43,44]. In addition, the perception of access, price, and supply of influenza vaccination also contribute to vaccine hesitancy [45,46]. Thus, PU and PEOU are important variables in assessing vaccine hesitancy. As mentioned above, research has confirmed that external factors like media communication can easily affect people’s awareness of the influenza vaccine and of vaccination. Therefore, it is feasible to apply the TAM as the theoretical framework in this study regarding its previous application and the characteristics of influenza vaccines.

Venkatesh, Viswanath, and Davis modified the original model and demonstrated that external variables would affect PU and PEOU, further changing a users’ intention to use technology and ultimately affecting their behavior [47]. This study defines media communication as an external variable to examine whether media information influences the PU and the PEOU of the influenza vaccine. Then, we investigate the willingness of the audience to use the influenza vaccine, that is, the intention to get vaccinated. In addition, it is difficult to track whether the research sample gets vaccinated in reality, so we ignore actual use variables in the TAM and define the intention to use as the outcome variable of this study.

### 2.2. Media Communication and Intention to Use

Concerning the broad nature of media communication, this study explores the influence of media communication on preventing influenza from the perspective of information recipients. From the view of information recipients, media exposure reflects how the audience receives information through various media, and media credibility shows how the audience comments on the received information. Furthermore, several studies have suggested that media exposure positively and significantly impacts media credibility [48,49,50]. Therefore, the audience’s media exposure should also affect media credibility regarding influenza prevention. Based on this, we propose the following hypotheses:

**H1:**  *In the communication of influenza information, media exposure significantly (positively) affects media credibility.*

Studies indicated that media exposure and credibility could influence people’s behavioral intentions [51,52]. Furthermore, media credibility can affect audiences’ health behaviors and decisions [53]. For example, a study of influenza vaccination in Canada during the 2009 H1N1 epidemic showed that media credibility is one factor affecting people’s intention to get vaccinated [54]. Accordingly, we hypothesize that:

**H2:**  *Media exposure significantly (positively) affects the public’s intention to receive the influenza vaccine.*

**H3:**  *Media credibility significantly (positively) affects the public’s intention to receive the influenza vaccine.*

### 2.3. Media Communication, Perceived Usefulness, and Intention to Use

In this study, PU refers to the extent to which survey participants believe that the influenza vaccine is beneficial to their health. In the TAM, PU is one vital variable that affects users’ intentions to use the vaccine. Based on the relevant literature and the actual situation of influenza vaccines, this study takes media exposure and media credibility as external variables. We posit that the public can perceive the usefulness of the influenza vaccine through media exposure, which improves the intention to get vaccinated. We proposed the following hypotheses based on the model:

**H4:**  *Media exposure significantly (positively) affects the public’s perceived usefulness of influenza vaccines.*

**H5:**  *Media credibility significantly (positively) affects the public’s perceived usefulness of influenza vaccines.*

**H6:**  *The public’s perceived usefulness of influenza vaccines significantly (positively) affects their intention to get vaccinated.*

### 2.4. Media Exposure, Perceived Ease of Use and Intention to Use

David pointed out that ease of use affects the user’s perceived usefulness of the new technology in TAM [55]. Specifically, the easier the system is to use, the more valuable it will appear to the user. Venkatesh and David defined PEOU as ‘the degree to which a person believes that using a particular system would be free of effort’ [56]. As the TAM model emphasizes users’ acceptance of new technologies, PEOU in influenza vaccination reflects the convenience, time, and effort required to get the influenza vaccine. Thus, we defined PEOU in this study as the survey participants’ assessment of how easy it is to receive the influenza vaccination. Combined with theoretical models, we proposed the following hypotheses:

**H7:**  *Media exposure significantly (positively) affects the public’s perceived ease of using influenza vaccines.*

**H8:**  *Media credibility significantly (positively) affects the public’s perceived ease of using influenza vaccines.*

**H9:**  *The public’s perceived ease of using influenza vaccines significantly (positively) affects their intention to get vaccinated.*

### 2.5. Perceived Usefulness, Perceived Ease of Use, and Intention to Use

It has been examined that PEOU positively influences PU in the TAM, affecting users’ intention to use the vaccine. Therefore, another hypothesis is proposed based on the TAM:

**H10:**  *The public’s perceived ease of using influenza vaccines significantly (positively) affects their perceived usefulness.*

## 3. Materials and Methods

### 3.1. Questionnaire Design

This study used the survey method to test the model. The questionnaire is divided into two parts. The first part measures the demographic characteristics of the participants with six items, including gender, age, region, monthly income, occupation, and education. The second part uses Likert scales to measure the four variables in the empirical model. Participants evaluate themselves about media exposure, perceived awareness of influenza prevention, and intention to get vaccinated. They specify the evaluations according to five points: strongly disagree, disagree, neither agree nor disagree (neutral), agree, and strongly agree.

There are three sources of measurement indicators in the questionnaire. First, we referred to the mature questionnaire scales from the relevant literature and modified them according to the actual situation. Second, we drew on relevant theories combined with comprehensive analyses from previous research. Third, we conducted a trial survey before the formal questionnaire, receiving a total of 132 valid trial questionnaires. Finally, we developed the questionnaire based on the results of the reliability and the validity tests and the feedback from respondents. Table 1 shows the definition of variables in the model, measurement constructs, and references.

### 3.2. Data Collection

We conducted the online survey via Tencent Questionnaire from 1 to 15 January 2022. Tencent Questionnaire is a free and professional questionnaire system launched by Tencent, and it is one of the most well-known online survey platforms in China. This platform provides different ways to start a survey, with simple and efficient editing methods and powerful logic settings. Tencent Questionnaire has served 22.697 million users, collecting approximately 2.72 billion questionnaires up to 19 March 2022 [60]. This study distributed questionnaires through the respondent groups in this platform. Tencent Questionnaire developed the respondent groups as a questionnaire distribution and answer function, and it helped to invite people who met the criteria to participate. Additionally, this function helped the members of the respondent groups to get opportunities to participate in different research. The groups in China have over one million participants, and every random respondent will receive CNY3.00 as a reward [60]. We distributed 658 questionnaires and gained 597 valid questionnaires after screening and eliminating irregular inquiries. The number of useful questionnaires is more than five times the total number of questionnaires, which meets the requirement of the survey method [61]. Table 2 shows the balanced distribution of participants according to demographic indicators, including gender, age, region, and education.

## 4. Results

### 4.1. Reliability and Validity Test

Table 3 introduces the final test results. According to SPSS 22.0, Cronbach’s alpha for the overall scale was 0.932. We designed the questionnaire based on the proposed hypotheses regarding content validity. We also modified the final questionnaire according to the feedback and the outcomes from the trial survey, which collected comments to understand the content. Besides, this study used SPSS 22.0 to test structural validity with the Kaiser–Meyer–Olkin Measure of Sampling Adequacy test (KMO value = 0.918) and Bartlett’s test of sphericity (*p* < 0.001). The test results indicated that factor analysis could be applied in this study.

### 4.2. Exploratory Factor Analysis

This paper adopted principal component analysis and variance maximum equilibrium rotation to conduct the factor analysis of five variables: media exposure, media credibility, perceived usefulness, perceived ease of use, and intention to use. The extracted common factors and the cumulative interpretation variance can be seen in Table 3.

According to the factor loading, the three factors obtained from the variable of media exposure were named as public and interpersonal communication factors (ME1), traditional media factors (ME2), and new media factors (ME3). The three factors obtained under the variable of media credibility were named as the new media factor (MC1), public and interpersonal communication (MC2), and traditional media (MC3). The four factors obtained from the variable of perceived usefulness were named as the priority for influenza vaccination (PU1), awareness of influenza (PU2), awareness of vaccine function (PU3), and effectiveness and risk of the influenza vaccine (PU4). Moreover, the two factors extracted from the perceived usefulness variable were named the vaccination cost (PEU1) and the vaccine supply (PUE2). The two factors obtained from the variable of intention to use were named ‘voluntarily get vaccinated’ (IU1) and ‘recommend getting vaccinated’ (IU2) (Table 3).

### 4.3. Confirmatory Factor Analysis

To further test the reliability and the validity of the scale, confirmatory factor analysis was performed on the measurement model. We calculated the standard load, average extraction variance (AVE), composite reliability (CR), and correlation coefficients between factors. The AVE value of each element was more significant than 0.5, indicating the scale’s good aggregation validity [62]. In addition to the CR value of PEOU (0.679), the values of other factors were all higher than 0.7, suggesting the scale’s excellent internal consistency [62]. As shown in Table 4, the square root of each factor’s AVE (value on the diagonal) was higher than the correlation coefficients, which means a good discriminant validity between the factors.

### 4.4. Model Analysis

**Figure 1:**
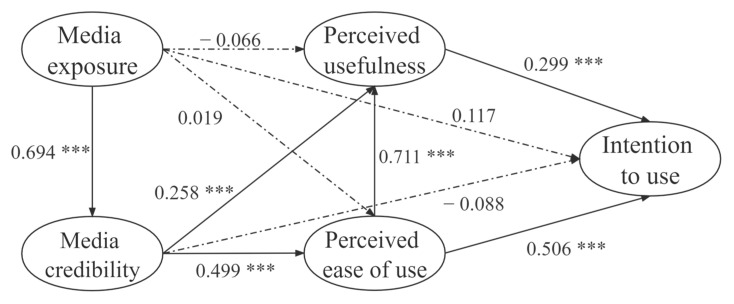
Results from the structural equation modeling procedure for the final model: values indicate standardized regression weights and the path load factor. Notes: *** *p* < 0.001, t > 3.29.

This research used AMOS17.0 for model analysis and modified the model through the modification index and the fitness index. The results showed that 6 of 10 hypotheses had significant results of *p* < 0.001 (H1, H5, H6, H8, H9, H10). Results with *p*-values over 0.05 indicated that the four hypotheses (H2, H3, H4, H7) were not supported. The explained variances of endogenous variables, including media credibility, perceived usefulness, perceived ease of use, and intention to use, were 48.2%, 70.6%, 26.2%, and 58.2%, respectively. Figure 1 is the schematic diagram for the structural equation model.

### 4.5. Model Evaluation

Model evaluation is generally operated with the model fitting index, which aims to measure the fitness of the collected data to the theoretical model. The model fitting indexes in this study were higher than the recommended index (X^2^/DF = 2.888, GFI = 0.962, AGFI = 0.935, NFI = 0.986, CFI = 0.994, IFI = 0.970, RMSEA = 0.054, RMR = 0.030), which proves that the proposed model fits the data well and the proposed model was established.

## 5. Discussion

### 5.1. Media Exposure and Intentions for Influenza Vaccination

This study identified that media exposure cannot directly influence the Chinese public’s intentions toward influenza vaccination, which differs from the findings of some related studies. For instance, Shropshire et al. found that mass media campaigns about improving influenza vaccination on campus increased vaccination coverage among university students [5]. Bonnevie et al. demonstrated that groups exposed to a flu vaccination campaign on social media were more likely to receive the influenza vaccination [63]. The difference can be explained from two aspects. On the one hand, the existing literature notably enhanced that influenza vaccination coverage is related to the number, timing, and promotion content of media releases. This paper had a different focus on the width and the frequency of media exposure rather than the usage of a specific media form. On the other hand, most of the previous research examined cases from countries outside of China, which have different media communication relative to China. In addition, this finding suggests that it is not enough to promote influenza vaccination to the public through media communication; other valuable factors must also be considered.

### 5.2. Media Credibility and Intentions for Influenza Vaccination

This paper examined that media credibility cannot directly influence intentions toward influenza vaccination, which is different from findings in previous research. For example, Burki researched the effect of media credibility on people’s intentions to receive influenza vaccination during the 2009 H1N1 pandemic in Canada, and it revealed that media trust is significantly important to vaccination attitudes. In particular, vaccine hesitancy occurred when respondents felt confused by media information [9]. Another research about the influence of media trust on COVID-19 vaccination argued that a high trust in traditional media decreased vaccine hesitancy and increased public motivation to receive COVID-19 vaccination [64]. However, this study indicated that media credibility could affect people’s intentions to receive influenza vaccination through PU and PEOU as PU and PEOU reflect public awareness of influenza, influenza vaccine, and influenza vaccination. Compared with media exposure, the public’s perception of the flu and its vaccine is more influenced by media credibility, when receiving relevant information. Participants in this study have more trust in professional medical institutions, traditional television media, and school health education institutions than other channels.

### 5.3. PU, PEOU, and Intentions for Influenza Vaccination

This study argued that PU could influence people’s intentions toward influenza vaccination. Nevertheless, the influenza vaccine is the non-National Immunization Program (NIP) vaccine in China, and the low coverage of non-VIP vaccination is related to a weak awareness of influenza and its vaccine [65]. For example, some people insist that children and the elderly don’t have to get an influenza vaccination [66,67], reflecting the public misunderstanding and the weak perceived usefulness of the benefits of influenza vaccination. A study on flu vaccine hesitancy showed that bias in the perceived risk and the perceived effects of influenza vaccine mainly contributes to influenza vaccine hesitancy [68]. Combined with the conclusions above, professional medical institutions, traditional television media, schools, and other channels with high media credibility can improve the public’s PU of the influenza vaccine through intensive and qualified communication.

In influenza vaccination promotion, PEOU shares the same importance as PU. As a non-NIP vaccine, the cost of and the access to the influenza vaccine also affect its vaccination coverage [65], which is also supported in this study. Furthermore, medical insurance in most regions of China does not cover the influenza vaccine, which needs to be given annually. Therefore, PEOU is essential to improve user experience and to promote vaccination. Specifically, PEOU refers to the location, cost, and availability of the influenza vaccine, affecting the public’s intentions to get vaccinated. Thus, we suggest releasing PEOU-related information via official and reliable media to promote influenza vaccination. In particular, information with public concerns, such as the appointment and vaccination price, deserves in-depth explanations.

### 5.4. Media Exposure and Media Credibility

This study showed that media exposure has a significantly positive influence on the public assessment of media credibility. Previous research also supported this finding and enhanced the significant role of credible media exposure in influenza vaccination promotion [48,49,50]. Increased exposure of the public to media allowed audiences to have the ability to assess influenza prevention information. With the assessment, the public built trust in certain media, which further influenced their preference in preventing influenza. 

According to the frequency of media exposure, the top five channels in this study where respondents received influenza information were social media, mobile applications, portals, interpersonal communication, and television. Social media and interpersonal communication have certain advantages over traditional media regarding media usage frequency. In this study, the top five influenza information channels with high media credibility among the public were professional medical institutions, television, school health education, grassroots organizations, and mobile applications. Compared with social media, health education from public institutions and television has more advantages.

On the one hand, although social media has shown absolute advantages in information dissemination based on new media technology, which indicates the issues in social media, such as a lack of scientific credibility, authenticity, and professionalism in communication. Studies also illustrated that social media brings risks such as providing low-quality information, violating personal and professional boundaries, and damaging the professional image of the field of health communication [69]. On the other hand, professional medical institutions, schools, and grassroots organizations have great media credibility when introducing influenza and influenza vaccine-related knowledge. A study also demonstrated that people prefer the disease prevention information released by authoritative, professional, and reliable media [70]. As mass media, television has been used to deal with public health emergencies for a long time, including epidemic knowledge popularization and disease prevention and control; thus, it has gained a high media credibility.

To summarize, media with great credibility is necessary for spreading influenza and influenza vaccine information, especially public institutions with authority and traditional media. Meanwhile, concerning the high exposure of social media, it is valuable to enhance the scientific, authorized, and professional content on these platforms, contributing to a higher media credibility and a stronger promotion of influenza vaccination.

### 5.5. Strengths and Limitations

This study indicated the role and the influence of the current media environment on public health. We investigated the influence mechanism of media on people’s intentions to receive influenza vaccination by introducing media exposure and media credibility and combining it with the TAM model. This study provided a new perspective for public health institutions to promote vaccination and to reduce vaccine hesitancy. However, this is an online survey, and the research sample has certain randomness. Additionally, the sample size of the priority groups for influenza vaccination was insufficient to carry out comparative research in this paper. Thus, future research will collect samples according to the distribution characteristics of China’s population structure to be more scientific.

## 6. Conclusions

This paper has argued that both media exposure and media credibility have no direct influence on the Chinese public’s influenza vaccination intention. However, as intermediate variables, perceived usefulness and ease of use allow media credibility to affect people’s intention to get vaccinated. In addition, this paper has proven that media exposure positively influences media credibility. The media with better credibility gained more trust from the audience, indicating a new perspective fin the promotion of influenza vaccination. Therefore, it is suggested to release influenza-related information via media with great credibility, further improving the public’s acceptance of vaccinations.

## Figures and Tables

**Table 1:** Definition of variables, measurement, and references.

Variables	Definition	Measurement	Number of Items	Reference
Media exposure	The extent to which users have encountered influenza-related information.	The frequency of reaching diverse media channels.	13	Lu and Andrews [57]
Media credibility	Users’ trust in the communication channels when receiving influenza-related information.	Trust evaluation of media channels that release influenza information.	13	Meyer [58]
Perceived usefulness	Users’ evaluation of the benefits of influenza vaccination.	Evaluation of influenza vaccine knowledge.	12	Davis [13] National Health Commission of PRC [59]
Perceived ease of use	Users’ evaluation of the ease of being vaccinated.	Evaluation of the place, cost, and availability of influenza vaccination.	3	Davis [13]
Intention to use	User intention to accept influenza vaccination.	Evaluation of intentions to voluntarily get vaccinated and recommendations to get vaccinated	3	Davis [13]

**Table 2:** Demographics of survey participants.

Variables	Items	Number	Percentage (%)
Gender	Male	299	50.1
	Female	298	49.9
Age	18–29	357	39.1
	30–39	154	23.4
	40–49	68	10.3
	50–59	15	2.3
	Over 60	3	0.5
Regions	Beijing, Shanghai, Shenzhen, Guangzhou	72	12
	Provincial capital cities and centrally-administered municipality (exclude Beijing, Shanghai, Shenzhen, Guangzhou)	125	21
	Prefecture-level cities	116	19.5
	Country-level regions	138	23.1
	Towns and villages	134	22.5
	others	12	2.0
Education	Middle school and under	98	16.4
	High school/technical secondary school/technical school	235	39.4
	Junior college	82	13.7
	Undergraduate	117	19.6
	MA and upper	65	10.9

**Table 3:** Exploratory factor analysis results.

Latent Variable	Extract Factor	Ingredients					Cumulative Interpretation Variance (%)	Cronbach’s Alpha
		1	2	3	4	5		
Media Exposure (ME)	ME1	0.864					58.249	0.864
	ME2	0.931						
	ME3	0.998						
Media Credibility (MC)	MC1		0.933				64.619	0.898
	MC2		0.858					
	MC3		0.782					
Perceived Usefulness (PU)	PU1			0.761			67.941	0.874
	PU2			0.839				
	PU3			0.775				
	PU4			0.712				
Perceived Ease of Use (PEU)	PEU1				0.979		81.693	0.688
	PEU2				0.811			
Intention to Use (IU)	IU1					0.866	90.344	0.835
	IU2					0.763		

**Table 4:** Confirmatory factor analysis.

Aggregation Validity Analysis					Discriminant Validity Analysis				
Latent Variables	Items	Loading	AVE	CR	ME	MC	PU	PEU	IU
MEDIA EXPOSURE (ME)	ME1	0.786	0.512	0.754	0.716				
	ME2	0.711							
	ME3	0.617							
MEDIA CREDIBILITY (MC)	MC1	0.689	0.568	0.798	0.559	0.754			
	MC2	0.841							
	MC3	0.738							
PERCEIVED USEFULNESS (PU)	PU1	0.732	0.506	0.801	0.295	0.477	0.711		
	PU2	0.618							
	PU3	0.663							
	PU4	0.829							
PERCEIVED EASE OF USE (PEU)	PEU1	0.748	0.515	0.679	0.276	0.386	0.574	0.718	
	PEU2	0.697							
INTENTION TO USE (IU)	IU1	0.793	0.692	0.818	0.283	0.35	0.548	0.594	0.832
	IU2	0.879							

## Data Availability

The datasets generated and/or analyzed during the current study are not publicly available due to the potential sensitivity of the content, but they are available from the corresponding author on reasonable request.
